# You are what you eat – The influence of polyphagic and monophagic diet on the flight performance of bees

**DOI:** 10.1002/ece3.70256

**Published:** 2024-09-02

**Authors:** Jula‐Klarissa Krüger, Sascha Buchholz, Sophie Schmitt, Katharina Blankenhaus, Nadja Pernat, David Ott, Hilke Hollens‐Kuhr

**Affiliations:** ^1^ Institute of Landscape Ecology, University of Münster Münster Germany; ^2^ Centre for Integrative Biodiversity Research and Applied Ecology University of Münster Münster Germany; ^3^ Centre for Biodiversity Monitoring and Conservation Science Leibniz Institute for the Analysis of Biodiversity Change Bonn Germany

**Keywords:** bumblebee, flight performance, physiological fitness, tethered flight (technique), unbalanced diet

## Abstract

Movement performance of insects is an important measure of physiological fitness and is likely affected by novel stressors associated with global change. Reduced fitness can lead to smaller foraging areas and thus to decreasing abundance, diversity and nutritional quality, which could weaken insect populations and contribute to global insect decline. Here, we combined two different methods: An experimental semi‐field design applying treatments in outdoor flight cages and a follow‐up experiment conducted in the laboratory, in which different parameters of movement performance, such as (a) velocity, (b) duration and (c) distance of an insect's flight can be quantified. We kept colonies of the bumblebee *Bombus terrestris* under contrasting nutritional conditions and measured treatment effects on the movement performance of individuals. Monophagously fed bumblebees showed reduced movement performance than polyphagously fed bumblebees. In particular, they stopped more frequently during flight, flew shorter distances and showed less often flight duration of 20 min. Our results suggest that nutritional deficiency due to a monophagic diet leads to reduced flight performance, which can have dramatic negative consequences for bees. Reduced flight performance may result in decreased availability of host plants, which may negatively affect stress resistance of bees and brood provisioning, facilitating extinction of insects. Although food of great nutritional value is an important compensator for the negative effects of different novel stressor, such as pesticides, it is not much known how to compensate for the effects of nutritional stress, especially in landscapes dominated by monocultures. However, our experimental approach with semi‐field and laboratory components has high potential for further studies investigating the impact of different stressors on the physiological fitness of insects but also body mass, or reproductive success and to find factors that may mitigate or even overcome the negative effect of stressors on insects.

## Graphical abstract

This study investigates the impact of nutritional conditions on the movement performance of bumblebees. Monophagously fed bees exhibited reduced flight performance compared to polyphagously fed bees, potentially leading to negative consequences for both bees and plants. The study highlights the importance of understanding how stressors like nutritional deficiency can affect insect fitness and ecosystem dynamics.

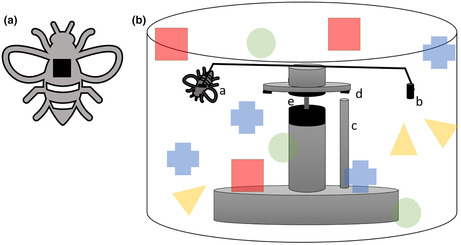

## 1 INTRODUCTION

Bees are known as the most important pollinators (Ballantyne et al., 2017) and are crucial for the sexual reproduction of many angiosperms and crops (Danforth, 2007; Klein et al., 2018). Due to climate change, anthropogenic land use change and the resulting fragmentation, as well as the increase in monocultures, many bee species, among other insects, are exposed to novel stressors such as insecticides, drought and or a less diverse flower supply (Hallmann et al., 2017; Hass et al., 2019; Sánchez‐Bayo & Wyckhuys, 2019; Straub et al., 2023). These novel stressors may have strong negative effects on the fitness of bees, for example, on sexual fitness, which potentially results in reduced reproduction rates, leading to the extinction of populations (Crall et al., 2018; Klaus et al., 2021; Ruedenauer et al., 2020; Straub et al., 2023). Furthermore, novel stressors may also have direct negative effects on the physiological fitness of bees, which potentially results in decreased body mass or reduced movement performance. Later, this results in a decline in the potential forage area of pollinators and consequently in a reduced availability (abundance, diversity and nutritional quality) of hostplants. This effect may further weaken the stress resistance as well as the sexual and physiological fitness of the pollinator population and foster the local extinction of insects (Kenna et al., 2019; Klaus et al., 2021; Tosi et al., 2017).

The importance of the topic has encouraged scientists to conduct research in this area, especially on the effect of heat and pesticides on bee fitness, whereas other stressors are still understudied. Straub et al. (2023) and Hass et al. (2019) tested the effect of land‐use‐associated stressors on pollinator health and colony growth. Heat stress also has a strong effect on bees as reviewed by Zhao et al. (2021), for example, negative effects on foraging behaviour (Souza‐Junior et al., 2020), development (Groh et al., 2004) or colony growth (Vanderplanck et al., 2019). Some studies have shown the negative effects of pesticides and insecticides on pollinators (reviewed in Raine & Rundlöf, 2024) particularly on pollinator mortality and lifespan effects (Manjon et al., 2018; Mundy‐Heisz et al., 2022), reproduction (Klaus et al., 2021), foraging behaviour (Arce et al., 2017; Stanley et al., 2016), nest behaviour (Crall et al., 2018), colony growth (Stanley et al., 2016) or thermoregulation (Tosi et al., 2017). Even the effect of multiple stressors, such as global warming in combination with the use of pesticides, on pollinator foraging behaviour, longevity or movement performance was tested (Albacete et al., 2023; Kenna et al., 2023).

Many of those novel stressors can be compensated by high‐quality nutrition, for example, heat stress (Vanderplanck et al., 2019) or insecticide effects (Klaus et al., 2021). However, this mechanism does not work in landscapes, dominated by monocultures, where the lack of a diverse flower supply causes nutritional stress for pollinators. (Straub et al., 2023; Vanderplanck et al., 2019): Bees forage on flowers for nectar, which provides them with carbohydrates, and pollen, which supplies them with nutrients such as proteins, lipids and micronutrients like sterols, minerals and vitamins (Grund‐Mueller et al., 2020; Roger et al., 2017; Roulston & Cane, 2000; Stabler et al., 2015; Wright et al., 2018). However, the pollen quantity and quality, particularly its nutrient composition, differs within and among plant species (Roulston & Cane, 2000; Ruedenauer et al., 2019). Hence, bees foraging on a less diverse flower supply may forage on inadequate or nutritionally inappropriate floral resources, which may weaken bees´ fitness. For example, Hass et al. (2019) found a positive correlation between pollen diversity and weight gain in bumblebee colonies. Furthermore, a low protein content (<37%) facilitated the offspring mortality rate of the wild bee species *Lassioglossum zephyrum* (Roulston & Cane, 2002) or a high content of lipids also has a negative effect on the sexual fitness of bumblebees (Ruedenauer et al., 2020). Nevertheless, it has also been shown that nutritional stress during the larval stage can be compensated (Wang et al., 2016). In contrast, Brodschneider et al. (2009) demonstrated that deficiencies, for example, smaller forewing and hindwing surface areas or reduced flight speed caused by artificially reared honeybee larvae, can only be partly or not at all compensated with adult nutrition. Consequently, there are still gaps in our knowledge of how nutritional stress is compensated and how this stress affects physiological fitness in the first place, for example, flight performance.

Flight performance has already been used as an indicator of physiological fitness and can be measured by characteristic variables such as speed, flight duration or stopping frequencies. Knauer et al. (2022) investigated the effect of multiple stressors, particularly nutritional stress and insecticides, on the flight performance of the wild bee *Osmia bicornis*. They measured offspring production, flight activity, flight duration and flower visitation frequency of the bees, which were held in flight cages with monocultures of either buckwheat, wild mustard or purple tansy. Tong et al. (2019) also researched the multiple effects of nutritional stress and pesticides on the flight success of honey bees (*Apis mellifera*). The bees were fed either an ad libitum sugar diet of rich (50% w/w sucrose solution) or poor (33%, leading to nutritional stress) quality and flight performance was measured with a flight mill (Tong et al., 2019).

In this study, we conducted a semi‐natural field experiment with bumblebee *Bombus terrestris* (hereafter simply bumblebees) as a model organism kept in flight cages. We exposed the bumblebees to different nutritional conditions consisting of monophagic and polyphagic diets that represented the landscape situation of large parts of the world, e.g., in central Europe or the United States. We used the plant species *Phacelia tanacetifolia* (high amount of nectar/flower and high protein content in pollen), *Centaurea cyanus* and *Sinapis arvensis* (both lower amount of nectar/flower and lower protein content in pollen) (Table S3). We offered either one plant species as a monophagic diet to create nutritional stress or a mixed culture of the three plant species as a polyphagic diet. In the lab, we investigated potential effects on flight performance under controlled conditions. In contrast to Knauer et al. (2022), who recorded the flight activity and length of nutritionally stressed bees by recording their entering and leaving the nest on video, we deployed a flight mill device to measure flight duration, flight distance and velocity (Kenna et al., 2019, 2021; Naranjo, 2019).

We expect monophagously fed bumblebees to have a worse flight performance than polyphagously fed bumblebees. A monophagic diet especially on *Centaurea cyanus* and *Sinapis arvensis* with a low amount of nectar/flowers and low protein content in pollen (Table S3) presumably results in an undersupply of nutrients, which negatively affects the bumblebee's metabolism and thus diminishes flight performance. We define flight performance as the interaction of the parameters (1) number of flight stops, (2) flight distance, (3) flight duration and (4) flight velocity. Specifically, we expect that polyphagously fed individuals of *Bombus terrestris* need (A) fewer flight stops and they fly (B) longer, (C) further and (D) faster than monophagously fed bumblebees.

## 2 METHOD

### 2.1 Field work

The experiment was conducted in the research garden of the University of Münster in North Rhine‐Westphalia, Germany (51°57′55.3″N, 7°36′22.2″O) from May to August 2021. Nine flight cages from Howitec Netting (7 × 3 × 2.5 m ORNATA PLUS) were setup in the field in two rows of four and five cages, respectively, with a minimum distance of 1.2 m (Figure S1). Each flight cage enclosed a raised bed (6 m length * 2.5 m width × 0.5 m height) filled with 30 cm topsoil divided into twelve 1 m^2^ plots. Each plot received 70 g of fertiliser (Blaukorn NovaTec, COMPO). Both rows contained randomly assigned monocultures, where either *Centaurea cyanus* L. (Cc), *Phacelia tanacetifolia* Benth. (Pt) or *Sinapis arvensis* L. (Sa) were planted in all plots, or mixed cultures comprising all three plant species. Thus, the study design contained six flight cages containing monocultures (two per plant species) and three mixtures. Pt and Cc are known as highly bee‐attractive species, whereas Sa is known to be less attractive for bees (BMEL – Bundesministerium für Ernährung und Landwirtschaft und Julius Kühn‐Institut, 2020). Seeds were purchased from Rieger Hofmann GmbH and planted on April 30, 2021.

In the mixed culture flight cages, the plots were sown randomly distributed across plots, receiving one out of the three plant species (intraspecific aggregation) per plot and using three plots per plant species to decrease interspecific competition (Waßmuth et al., 2009). Three plots remained unplanted in each mixed culture flight cage. Per plot, 0.5 g seeds of Cc and Sa and 2 g seeds of Pt were evenly spread on the topsoil. Plants were watered as needed and unsown plants were weeded. The flower cover was measured weekly in quartiles (0–25%, 25–50%, 50–75%, 75–100%). As soon as the flower cover in a mesocosm reached the third quartile (50–75%), which happened in all mesocosms in June 2021, one colony of the bumblebee *Bombus terrestris* of standard age and size per mesocosm was ordered by commercial providers (Natupol Smart, by Hummelvertrieb Sven Behr & Koppert Biological Systems). In total, we ordered nine colonies, one colony for each mesocosm. After bumblebee colonies arrived, they were setup in the flight cages upon a small box to avoid ground‐level contact. The box was painted with insect glue to prevent ants from entering the colonies. The colonies were protected against rain and direct sunlight with a self‐built shelter. The colony nests also contained a bag of sugar solution, which was freely accessible during the entire experiment. Bumblebee access to flight cages had no time restrictions. To validate that bumblebees forage polyphagously in the mixed culture flight cages, we observed four marked individuals between 13 and 60 min in July 2021. We found that bumblebees visited different flower species during their foraging trips similar to Martínez‐Bauer et al. (2021) (Figure S2).

### 2.2 Flight mill

Movement performance was investigated using flight mills (Figure S3). In principle, a flight mill is based on magnetic levitation technology, and it measures the linear velocity, distance, and duration of an insect's flight when tethered to the device. See corresponding technical protocols (reviewed by Naranjo, 2019) and available instructions and designs (e.g. by Smith & Jones, 2012), but see also Attisano et al. (2015), Kenna et al. (2019) or Tosi et al. (2017) for further information on the device. The basic flight mill consists of three parts: a base at the bottom, a middle part built with two plastic cylinders, two magnetic rings, an injection needle, a disc and on the top a flight arm made out of an injection tube (Figure S3). A plastic cylinder is attached to the stand, and a magnetic ring is fixed to the upper end of this plastic cylinder. An injection needle in the centre of the plastic cylinder serves as an axial connection to a second upper plastic cylinder. The upper cylinder is provided with an opposite magnetic ring at the lower end, in the middle part is a disc with two opposing magnets and a 32 cm long injection tube passes horizontally through the upper end, which acts as the flight arm. On both ends of the flight mill's arm, a magnet is installed, one for attaching the insect and one for attaching a counterweight (Figure S3; Video S1). The arm is removable from the construction, which eases the process of tethering the insect (Video S1). A Hall effect sensor is installed on the site of the lower plastic cylinder and is triggered by the two opposing magnets on the disk (Figure S3). The sensor sends the signal to a computer (we used Microsoft Windows 10) by using an Arduino UNO R3‐microcontroller. The driver CH341SER from the company WCH was used to control the microcontroller (WinChipHead, 2021) and the software program Cleverterm‐2.4.4 read and saved the data (Bürmann, 2021).

The Arduino microcontroller was programmed to measure the time between two signals from the sensor of the flight mill. This timespan corresponds to the travelled distance between the two magnets, which is half a circuit. The measured time of two following signals is then automatically counted by the microcontroller to yield a measure of a full circuit. Knowing the size of the circuit (in our apparatus, the circumference at the end of the flight arm is 1 m (U = π * 32 cm)), the gathered measure of distance transfers to distance in meters. Subsequently, linear velocity is calculated in meters per second. In a nutshell, the software records the linear velocity (meters per second) and the number of arm rotations per experiment.

The flight mill was protected against the wind with a transparent cylinder (diameter: 46 cm; height: 40 cm) made of a flexible plastic sheet. The cylinder serves as a visual cue for flying insects by colouring it with different symbols and colours (in our case study: four blue crosses, three red triangles, three green circles and three yellow triangles covering an area of 13%) (Minter et al., 2018; Naranjo, 2019) (Figure S3; Video S1).

### 2.3 Bee tagging

The tagging was carried out in two rounds. The first tagging round was conducted three to 7 days after the arrival of bumblebees in the cages, 20 individuals per cage were tagged. The second tagging round was done 15 to 19 days after arrival; again, 20 individuals per cage were tagged (Table S4). For tagging, bumblebees were collected directly from flowers in the flight cages (using commercially available bee catcher clips by Zerodis) to ensure that these are actively collecting workers who also appear to be healthy for the tagging process (Tosi et al., 2017). Individuals were kept in a small mesh cage provided with flowering plants and 50% sucrose solution. The tagging process was done in the field using a camping table in a wind and sun shelter spot. Individuals were immobilised for tagging (Hagen et al., 2011; Naranjo, 2019) by sedation with CO_2_, which is a common approach for sedation of insects (Naranjo, 2019). Therefore, each individual was taken out of the mesh cage one by one and stored in a beaker closed with foam material. CO_2_ was injected into the beaker until the individual stayed motionless. The individual was taken out of the beaker immediately to avoid asphyxiation. A small tag (ferrous metal plate or a magnet, e.g., cut from magnetic tape) was glued to the centre of the thorax using tweezers and UV glue (UHU LED‐Light Booster) (Video S2). The used magnets had a diagonal length of 3.5 mm and a mean weight of 24 mg (±2 SD, *n* = 10), which is 7.7% of the mean body mass of the investigated bumblebees (313 mg ± 6 SE, *n* = 229). The magnets were marked with different colours using nail polish to distinguish between different days of tagging. The tagged bumblebees were taken back into the flight cages to recover from the treatment. After a minimum of 48 h or a maximum of 14 days (on average 7 days) of recovery, the tagged individuals were recaptured for the flight mill experiment. For each flight mill experiment event, six individuals per flight cage were recaptured. Table S4 shows a detailed plan of when the bees were tagged and when the corresponding flight mill experiment took place. The recapturing rate of individuals equipped with tags averaged around 58% across all treatments (Table S1). The recaptured individuals were transferred into a mesh cage and taken to the laboratory for the flight mill experiment, ensuring no exposure to extreme weather conditions (such as wind chill, direct sunlight or raised temperature) and in standardised transfer time (approx. 20 min).

### 2.4 Flight performance measures

The measures took place during the daytime (9 am – 5 pm) and warm room temperatures (27°C ± 2°C). In the laboratory, each individual was transferred into a small beaker closed with cotton wool. The beaker was weighed with and without the bumblebee using a semi‐micro precision scale (Satorius SECURA225D‐1S) to achieve the live body mass. Counterweights (small balls weighing either 200, 250, 300 or 350 mg made from modelling clay) were chosen to balance the weight of the bumblebees. The counterweight was magnetically attached to the end of the flight arm opposite to the bumblebees to ensure levelling of the flight arm. The inner side of the cotton wool stuffing the beakers was soaked with 50% sucrose solution and offered to the bumblebee immediately before the experiment started. Before the tethering process, bumblebees were examined in a health check to control for damaged wings or parasites and exclude such unhealthy individuals. The tag position was described using the categories from Kenna et al. (2019) (ideal, unideal, unacceptable). After tethering bumblebees to the flight arm of the flight mill, the bees spent a short acclimatisation period of up to 3 min on a landing platform built out of a toilet paper roll and a petri dish. Since the magnets only have low magnetic force, insects were able to rotate around themselves when they were attached to the flight mill, despite the magnet, achieving near‐natural angles of flight in the circuit. If the bumblebee individual did not start flying spontaneously, the platform was turned until the insect was orientated into flight direction, and then the platform was removed quickly to stimulate flight (Kenna et al., 2019). If the stimulation was not successful, the legs of the bumblebee were carefully touched up to three times with the landing platform and released again. If the bumblebee did still not start flying, it was excluded from the experiment. Otherwise, a number of stimulations to initiate flight as well as a number of stops during flight were noted. In case the bumblebees quit flying, the platform was first offered again for resting for 30 s and subsequently removed again up to three times to stimulate flight. The flight performance of individuals was measured in experimental runs standardised to a maximum of 20 min. We choose a maximum of 20 min to be able to test more bumblebees during the experiment. Furthermore, the experiment was finished when the bumblebee stopped its flight for the fifth time, similar to Kenna et al. (2019). In order not to distress – the individuals who did not want to fly and constantly aborted the flight, we defined the cut‐off at 5 stops to be sensible. These restrictions (time limit of 20 min and 5 flight stops as maximum) must be considered when interpreting the data.

A data collection protocol is provided in the Table S2.

### 2.5 Data filtering and analysis

Before data analysis, the data collected with the flight mill were filtered similar to Kenna et al. (2019). The first flown circuit after a flight stop was deleted since the velocity is often extremly high in the first circuit compared to the following circuits due to stimulatory stress. Furthermore, the final three circuits before an individual terminates its flight were also deleted, because stopping the wing movements does not lead to an abrupt halt of the flight mill but to a gradual slowing down.

With this filtered data set we calculated three parameters as a measure of flight performance: (1) we summed up the number of flight stops, (2) calculated the total distance (number of flown circuits * 1 m – circumference of flight mill arm), (3) total flight time as the sum of individual circuit times, and (4) the mean velocity as the average taken all circuit velocities into account and noted the maximum flight velocity for each individual.

All statistical analyses of the data were conducted in RStudio (R Core Team, 2020). To test differences in flight performance traits, two levels were considered: first level: polyphagous versus monophagic diet and second level: polyphagic diet versus all plant species separately (Cc, Pt, Sa). After data inspection, for the first test level, either a *t*‐test was used for normally distributed and variance homogenous data, whereas a Mann–Whitney‐U‐Test was used if the data were not normally distributed. For the second test level, an ANOVA was used for normally distributed and variance homogenous data or Kruskal‐Wallis‐Test for not normally distributed data and variance homogenous or heterogenous data, followed by Post‐hoc‐Tests. A generalised linear mixed model (GLMM) was applied to investigate if the colony has an effect on the relationship between alive body mass (predictor) and flight performance (response variables since the colony had no effect, a generalised linear model (GLM) was applied to investigate the impact of alive body mass (predictor) on the flight performance (response variables): number of flight stops as relative data (binomial), mean velocity (Gamma), total duration as relative data (binomial), total distance (Poisson)).

## 3 RESULTS

### 3.1 Condition of tagged bumblebees

In total, 238 bumblebees (a minimum of 20 individuals and a maximum of 30 individuals per cage) were recaptured for the flight mill experiment, whereas nine bumblebees showed technical problems like they either lost their magnet when connecting it on the flight mill or the polarity of the magnet on the thorax was not correct and they were excluded from the experiment (Table S1). Out of the 229 remaining bumblebees, 223 individuals initiated flight, whereas six individuals did not perform. Regarding health problems, 17 bees had light damage on their wings; however, no significant differences were found in flight performance between individuals that had damaged and no damaged wings (Table S1). Furthermore, there were no bees with unacceptable tag positions, 200 individuals were tagged ideally, and 23 individuals had unideal tag positioning (tag not centred on the thorax). However, there were no significant differences in flight performance between bumblebees with tag position ideal or unideal (Table S1).

### 3.2 Wet mass of tagged bumblebees

There were no significant differences in wet mass on the first level, particularly between monophagously and polyphagously fed bumblebees (Mean_Mono_ = 323.4 mg ± 10.6, Mean_Poly_ = 300.3 mg ± 7.4: Kruskal‐Wallis: chi‐squared = 3.396, df = 1, *p* = .065). At the second level, we found significant differences: bees that fed on Sa (mean = 364.4 mg ± 12.78 SE) had significantly more wet mass compared to bees that fed on Cc (mean = 287.3 mg ± 10.27 SE) or on the mixed culture (mean = 300.3 mg ± 7.4) (Kruskal‐Wallis: chi‐squared = 21.168, df = 3, *p* < .001; Post‐hoc Sa versus Cc: *p*‐adjusted <.001; Post‐hoc Sa versus polyphagic diet: *p*‐adjusted = .002).

### 3.3 Flight performance

#### 3.3.1 Flight stops

**FIGURE 1:**
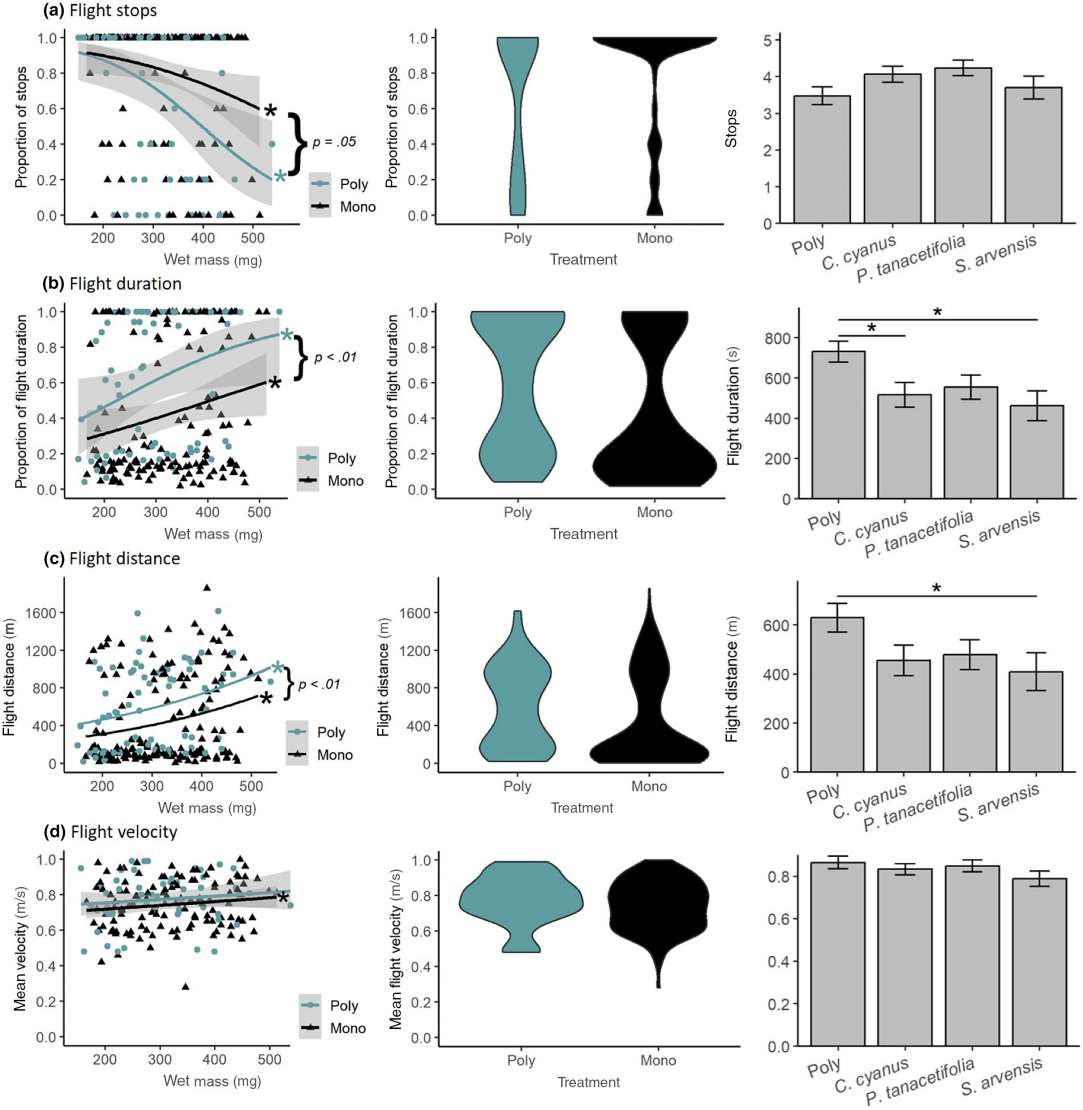
Flight performance results. The y‐axis of the parameter flight stops and flight duration is scaled in proportions since these parameters had a cut‐off in the experiment either after 5 flight stops [5 stops = 1 (100%, specified maximum of stops)] or 1200 s [1200 s = 1 (100%, specified maximum of flight duration)]. Significant increases or decreases between flight performance parameters and wet mass are marked with symbols using * (left graph). Significant differences between monophagaously and polyphagously fed bumblebees are shown by the *p*‐value (left graph). The frequency distribution of polyphagously or monophagously fed bumblebees for each flight performance parameter is shown with violin plots (middle plots). Significant differences at level two between monophagously fed bumblebees and feeding on single plant species are marked with symbols using * (right graph).

Bumblebees with a monophagic diet had on average 3.5 stops per experiment, significantly less than bumblebees with a polyphagic diet (mean = 4 stops; chi‐squared = 4.0777, df = 1, *p* = .045) (Figure 1a). Of the monophagously fed bees, 73% had five stops, but only 60% of the polyphagously fed bees stopped five times (Figure 1a). Only 9% of the monophagously fed bees did not abort flight once during the experiment in contrast to 16% of the polyphagously fed bees (Figure 1a). There were no significant differences at the second level (monophagic diet versus each plant species: Kruskal‐Wallis: chi‐squared = 5.189, df = 3, *p* = .1585) (Figure 1a). Both monophagously and polyphagously fed bumblebees showed a significant negative relationship between wet body mass and a number of flight stops (GLM_Poly_: *z* = −2.919, *p* = .003, GLM_Mono_: *z* = −2414, *p* = .016) (Figure 1a).

#### 3.3.2 Flight duration

Mean flight duration for polyphagously fed bumblebees was significantly longer compared to monophagously fed bumblebees (Mean_Poly_ = 731 s ± 53 SE; Mean_Mono_ = 513 s ± 37 SE; chi‐squared = 14.195, df = 1, *p* < .001) (Figure 1b); particularly bumblebees that fed on Sa or Cc only showed less flight duration (Kruskal‐Wallis: chi‐squared = 17.141, df = 3, *p* < .001; Post‐hoc Sa: *p*‐adjusted = .004; Post‐hoc Cc: *p*‐adjusted = .012) (Figure 1b). There was a positive significant relationship between flight duration and wet body mass (GLM_Poly_ *z* = 2.029, *p* = .04, GLM_Mono_: *z* = 1.982, *p* = .048) (Figure 1b). The maximum flight duration of 1200 s (20 min) was reached by 49.3% of polyphagously and 32.9% of monophagously fed bumblebees (Pt = 37.3%, Cc = 31.6%, Sa 29.6%) (Figure 1b).

#### 3.3.3 Flight distance

Polyphagously fed bumblebees flew on average significantly farther than monophagous fed bumblebees (Mean_Poly_ = 630 m ± 58 SE; Mean_Mono_ = 451 m ± 38 SE; chi‐squared = 10.851, df = 1, *p* < .001) (Figure 1c). Especially bumblebees that foraged monophagously on *Sinapis arvensis* flew only on average 409 m ± 77 SE (Kruskal‐Wallis: chi‐squared = 14.023, df = 3, *p* = .003; Post‐hoc Sa: *p*‐adjusted = .007) (Figure 1c). Wet body mass of monophagously fed bumblebees was significantly positivly related to flown distance (GLM_Mono_: *z* = 57.78, *p* < .001). This trend is also indicated for polyphagously fed bumblebees; however, it was just not significant (GLM_Mix_: *z* = 36.45, *p* < .001) (Figure 1c).

#### 3.3.4 Velocity

The mean and maximum velocity of monophagously and polyphagously fed bumblebees was similar (Mean_Poly_ = 0.86 m/s ± 0.030 SE, Mean_Mono_ = 0.83 m/s ± 0.017 SE, chi‐squared = 1.274, df = 1, *p* = .260; Max_Poly_ = 1.56 m/s ± 0.11 SE, Max_Mono_ = 1.55 m/s ± 0.09 SE, chi‐squared = 0.74168, df = 1, *p* = .390) (Figure 1d). There were also no differences in mean and maximum velocity on the second level considering polyphagous died versus each plant species separately (Polyphagous versus each plant species = Kruskal‐Wallis_Mean_Velocity_: chi‐squared = 3.6131, df = 3, *p* = .301; Kruskal‐Wallis_Max_Velocity_: chi‐squared = 2.576, df = 3, *p* = .462) (Figure 1d). Likewise, no relationship between wet body mass and mean or maximum velocity could be found (GLM_Mean_Velocity_Poly_: *t* = 0.0954, *p* = .924, GLM_Mean_Velocity_Mono_: *t* = 2.32, *p* = .022; GLM_Max_Velocity_Poly_: *t* = 0.561, *p* = .5774, GLM_Max_Velocity_Mono_: *t* = 0.012, *p* = .99) (Figure 1d).

## 4 DISCUSSION

To our knowledge and in relation to Kenna et al. (2019), Knauer et al. (2022) and Tong et al. (2019), this study is one of the first to investigate the effects of the scarce nutritional situation, mimics a landscape dominated by monocultures with a few mixed crops, on the flight performance of bees. Our data showed that velocity was not affected by the diet, which is in line with the results of Ellington et al. (1990), who showed that the metabolic rate of bumblebees is relatively stable with velocity ranging between 0 m/s and 4 m/s. However, flight cage size and optic flow (magnitude of apparent image motion) might have a strong effect on the velocity of flying insects (Baird et al., 2010; Dyhr & Higgins, 2010; Linander et al., 2016). Bumblebees held in smaller flight cages (120 * 100 * 35 cm) fly slower (~0.2 m/s) (Spaethe et al., 2001) than bumblebees in nature (3–15 m/s) (Osborne et al., 1999). In addition, bumblebees flew significantly faster in wider tunnels since with increasing distance between walls the axial optic flow cues decreased and bumblebees dared to increase their velocity (Baird et al., 2010; Dyhr & Higgins, 2010). However, in contrast to velocity, all other parameters of flight performance showed significant differences between bees that fed on monophagous or polyphagic diets, confirming our expectations. Particularly bumblebees that fed on different flower species needed less flight stops and flew longer and further.

The monophagously fed bumblebees, especially when they foraged on *Sinapis arvensis* only, showed the poorest flight performance. Flight performance can be influenced by both the energetic metabolism and the physiological constitution of bumblebees: Their energetic metabolism is mainly driven by the consumption of nectar, which provides the energy for flight through carbohydrate oxidation (Suarez et al., 1996, 2005). In our study, *Sinapis arvensis* has the lowest nectar amount per flower compared with the other studied plant species (Filipiak et al., 2022) (Table S3). It likely delivers less energy for flight activities potentially resulting in reduced flight performance. However, in our experiment, the bumblebees were offered 50% sucrose solution before they were connected to the flight mill to ensure a good energy supply. Hence, the reduced physiological constitution of monophagously fed bumblebees compared to polyphagously fed bumblebees is likely the reason for the reduced flight performance of monophagously fed bumblebees. The physiological constitution of bumblebees is mainly driven by the quality of foraged pollen, the main resource for lipids, proteins, amino acids, vitamins and minerals for bumblebees. A diet with low pollen quality leads to smaller bodies for *Lassioglossum zephyrum* and carpenter bees (Lawson et al., 2021; Roulston & Cane, 2002), decreased honey bee physiology (Di Pasquale et al., 2013), or weakened health state of bumblebees (Roger et al., 2017; Straub et al., 2023). Vanderplanck et al. (2014) even found that insufficient nutrition already negatively impacts the growth of bumblebee larvae. Our results fit in well with these results: bumblebees that fed on *Sinapis arvensis* or *Centaurea cyanus* only, both having the lowest protein contents (Filipiak et al., 2022; Roulston & Cane, 2000) (Table S3), showed lower flight performance.

The negative effects of some novel stressors can be compensated by certain environmental conditions. Heat stress can be compensated, for example, by thermoregulation of the colony when bees actively ventilate their nest by wing fanning (Maebe et al., 2021; Westhus et al., 2013) or by adjusting their foraging activity time to the morning or evening (Maebe et al., 2021; Stelzer et al., 2010). The effect of heat stress on bees can also be mitigated by high‐quality nutrition (Vanderplanck et al., 2019). The use of pesticides is also an effective novel stressor for bees, but it can also be compensated to a certain extent. For example, by an increase of natural habitats such as forests, wooded and herbaceous wetlands, shrublands and grasslands in the surrounding landscape (Park et al., 2015) or by high‐quality nutrition as shown by Klaus et al. (2021) or Rundlöf et al. (2022) for wild bees, or by Zhang et al. (2023) and Castle et al. (2022) for honey bees, respectively. But in the intensively farmed landscapes of central Europe and the USA, with large areas of monoculture, the supply of food with high nutritional value continues to decline (Filipiak et al., 2022; Lau et al., 2023), depriving pollinators of the opportunity to mitigate novel stressors with high‐quality nutrition.

So far, little is known about the ways wild bees react to and compensate for the nutritional stress in these areas. Insects may balance their nutrient needs by consuming additional resources, and therefore, expand their foraging radius to achieve and maintain optimal nutrient supply (Redhead et al., 2016; Vaudo et al., 2016). However, our results indicate that this can be a vicious cycle for bumblebees, particularly in landscapes dominated by monocultures. On the one hand, the monophagous food supply forces bees to expand their flight radius to achieve additional resources, on the other hand, it reduces their flight performance, which minimises the flight radius and hence accessibility of additional resources. This constraint likely decreases the survival, reproduction and stress resistance of bee populations (Baloglu & Gurel, 2015; Hass et al., 2019; Klaus et al., 2021; Vaudo et al., 2015).

In contrast, a landscape with a diverse range of flowers, reflected by the mixed plants of our experiment, provides bumblebees with mixed pollen (polyphagic diet) and facilitates a more balanced diet and thus flight performance. Our findings complement the results of previous studies showing a positive correlation between floral diversity and fitness, for example, measured as colony weight and colony growth (Goulson et al., 2002; Hass et al., 2019; Kämper et al., 2016). Therefore, in order to support bee populations in the landscape, ensure the ecosystem service of pollination in the future and reduce pollinator decline, an increase and diversification of the flower supply in agricultural landscapes is required (Klaus et al., 2021; Rundlöf et al., 2022; Straub et al., 2023). Due to the large differences in the qualitative composition of the plants (Filipiak et al., 2022; Roulston & Cane, 2000), it is important not to plant many different plants at random, but to ensure that all the necessary nutrients are covered when selecting plant species e.g. for flower strips (Castle et al., 2022). For this purpose, further plants, native species as well as neophytes, should be tested to investigate their effects on the fitness of pollinators – as a monoculture but also in mixed cultures. Furthermore, when selecting suitable plants, it should also be taken into account that the supply of nutrients is guaranteed throughout the entire activity period of bees, as suggested by Lau et al. (2023).

High‐quality nutrition appears to be a pivotal point for healthy bee populations but is part of a conundrum due to its limited availability in modern agricultural landscapes. Although food of great value is an important compensator for the negative effects of novel stressors (Klaus et al., 2021; Rundlöf et al., 2022; Vanderplanck et al., 2019; Zhang et al., 2023), when it is unavailable, the effects of, for example, pesticides are exacerbated (Knauer et al., 2022; Tong et al., 2019). Hence, we want to highlight that there is a demand for further research on how nutritional stress can be compensated, especially in landscapes dominated by monocultures. Of the many novel stressors to consider, many studies have focused on heat (Kenna et al., 2021; Souza‐Junior et al., 2020; Vanderplanck et al., 2019) or pesticides (Albacete et al., 2023; Kenna et al., 2019; Manjon et al., 2018; Mundy‐Heisz et al., 2022; Park et al., 2015; Raine & Rundlöf, 2024; Stanley et al., 2016), whereas similar important stressors like increasing CO_2_ concentration (Otieno et al., 2022) or microplastics (Ferrante et al., 2024) are rarely investigated. Our approach to associate fitness, approximated by flight performance, with nutritional stress is easily transferable to a wide range of plant species, pollinators and novel stressors. Results from these experiments will not only help to identify performance thresholds and mitigating or even neutralising factors for negative effects but also to understand the causes of recent insect declines and are, therefore, an important contribution to species conservation.

## AUTHOR CONTRIBUTIONS

**Jula‐Klarissa Krüger:** Investigation (lead); methodology (lead); writing – original draft (equal). **Sascha Buchholz:** Formal analysis (equal); supervision (supporting); validation (equal). **Sophie Schmitt:** Investigation (supporting); methodology (supporting); writing – original draft (supporting). **Katharina Blankenhaus:** Investigation (supporting); methodology (supporting); writing – original draft (supporting). **Nadja Pernat:** Writing – original draft (supporting). **David Ott:** Conceptualization (equal); funding acquisition (supporting); supervision (supporting). **Hilke Hollens‐Kuhr:** Conceptualization (equal); funding acquisition (lead); investigation (supporting); supervision (lead); visualization (lead); writing – original draft (equal).

## FUNDING INFORMATION

None.

## CONFLICT OF INTEREST STATEMENT

The manuscript describes original work which has not been published previously and is not under consideration by any other journal. All authors approved the manuscript and this submission. Authors have no actual or potential conflict of interest or competing interest, including any financial, personal or other relationships with other people or organisations that could inappropriately influence or be perceived to influence our work.

## Supporting information


Video S1:

Video S2:

Data S1:

## Data Availability

All data are published in the manuscript. If desired, the data can also be made available at dryad.
