# Supplementary material for: You are what you eat – The influence of polyphagic and monophagic diet on the flight performance of bees
Source: Ecol Evol. 2024 Sep 2;14(9):e70256. doi: 10.1002/ece3.70256 (PMC11368496; doi:10.1002/ece3.70256)
Supplement: Supplementary file 3 — Data S1: [file ECE3-14-e70256-s002.docx]

Supplementary Information to manuscript

You are what you eat - the influence of polyphagous and monophagous diet on the flight performance of bees

# Supplementary Figures

## Supplementary Figure 1

##
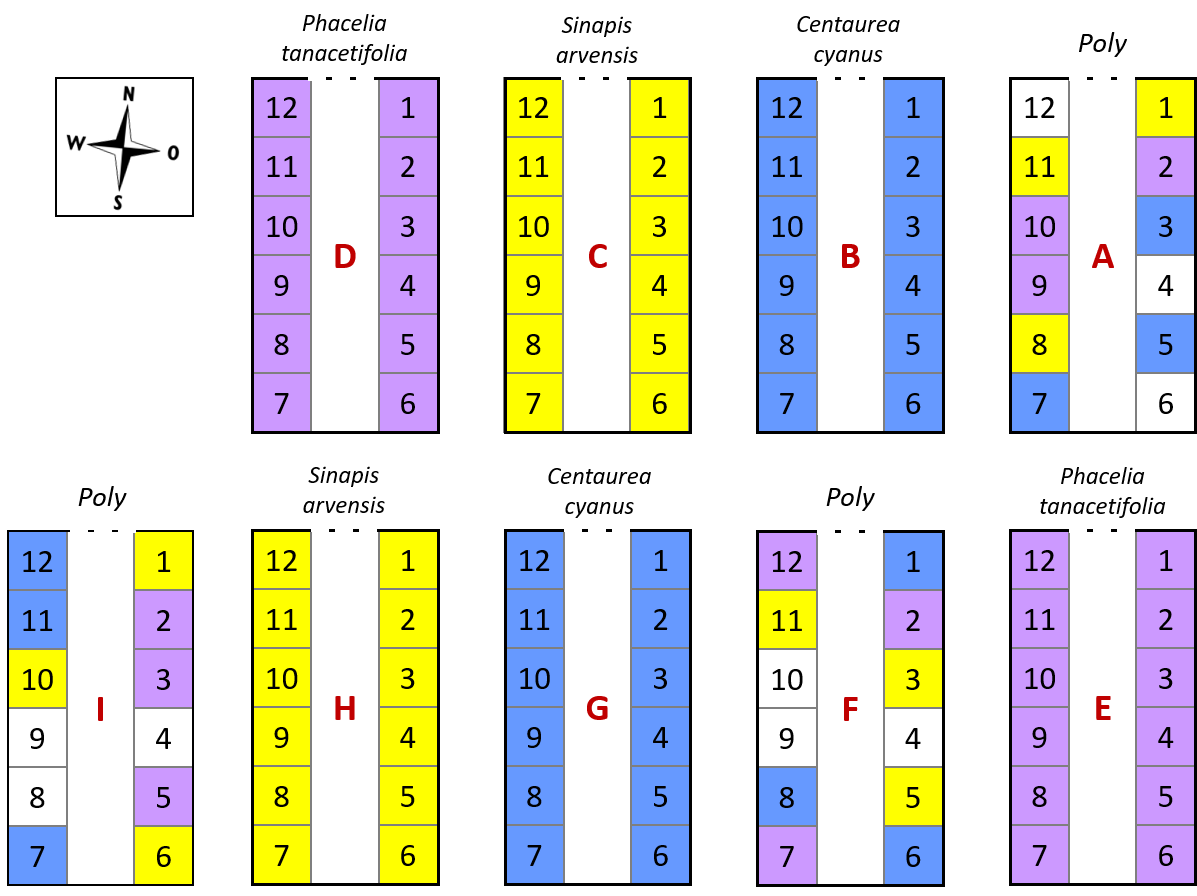


Supplementary Figure 1: Set up of flight cages in the field. Flight cages contained either monocultures (B, C, D, E, G, H,) or mixtures (A, F, I) and were divided into twelve 1 m² plots. Plots were planted with either *Phacelia* *tanacetifolia* (coloured in purple), *Sinapis* *arvensis* (yellow), *Trifolium* *pratense* (red) or *Centaurea* *cyanus* (blue), unplanted plots (white).

## Supplementary Figure 2


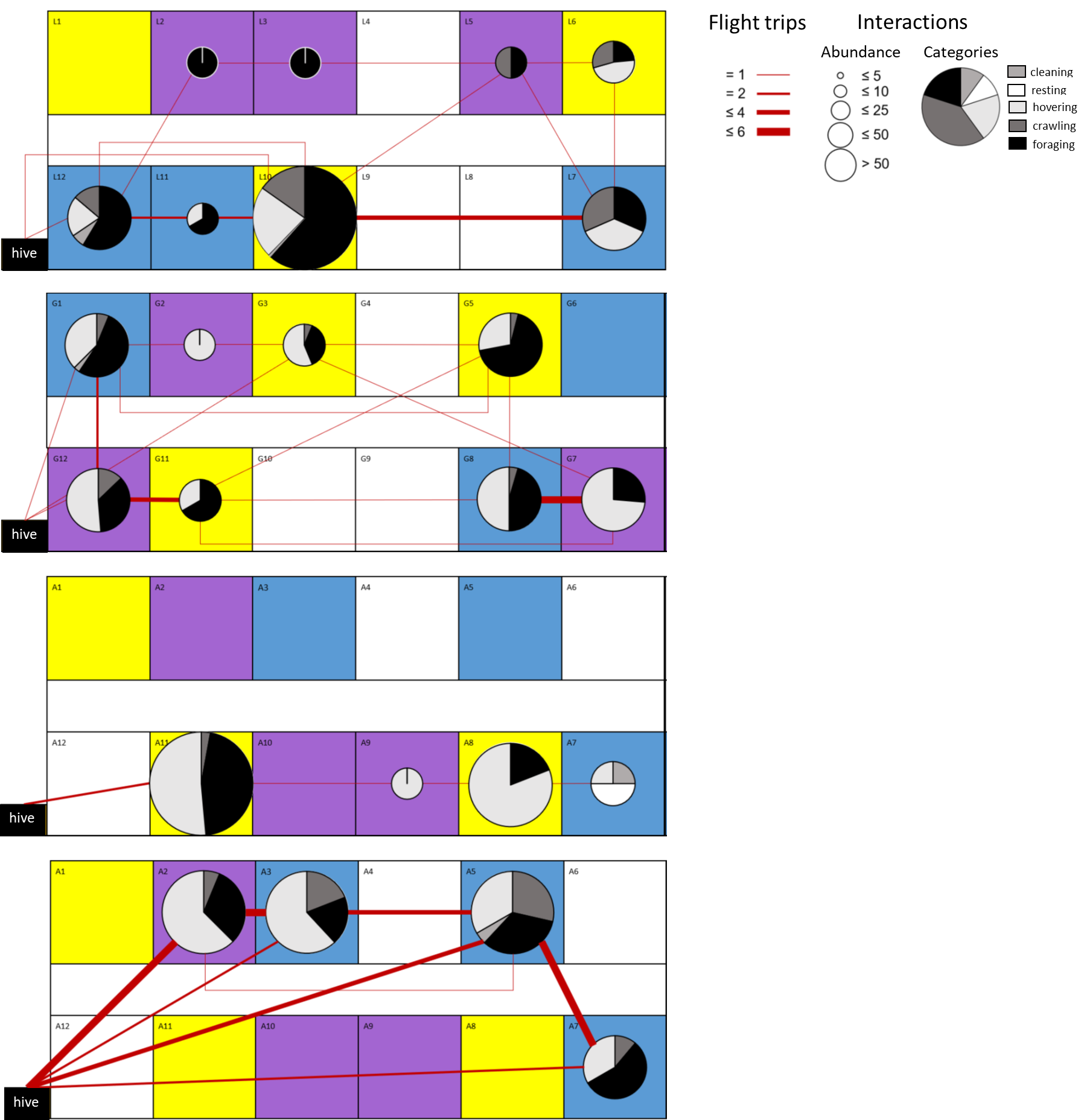


Supplementary Figure 2: Observations of four marked bumblebees in flight cages with mixed plant species (Yellow plots = *Sinapis arvensis*, violet plots = *Phacelia* *tanacetifolia*, blue plot = *Centaurea cyanus*). Pies are showing abundance and kind of interaction on hostplants, red lines show number of flight trips.

## Supplementary Figure 3


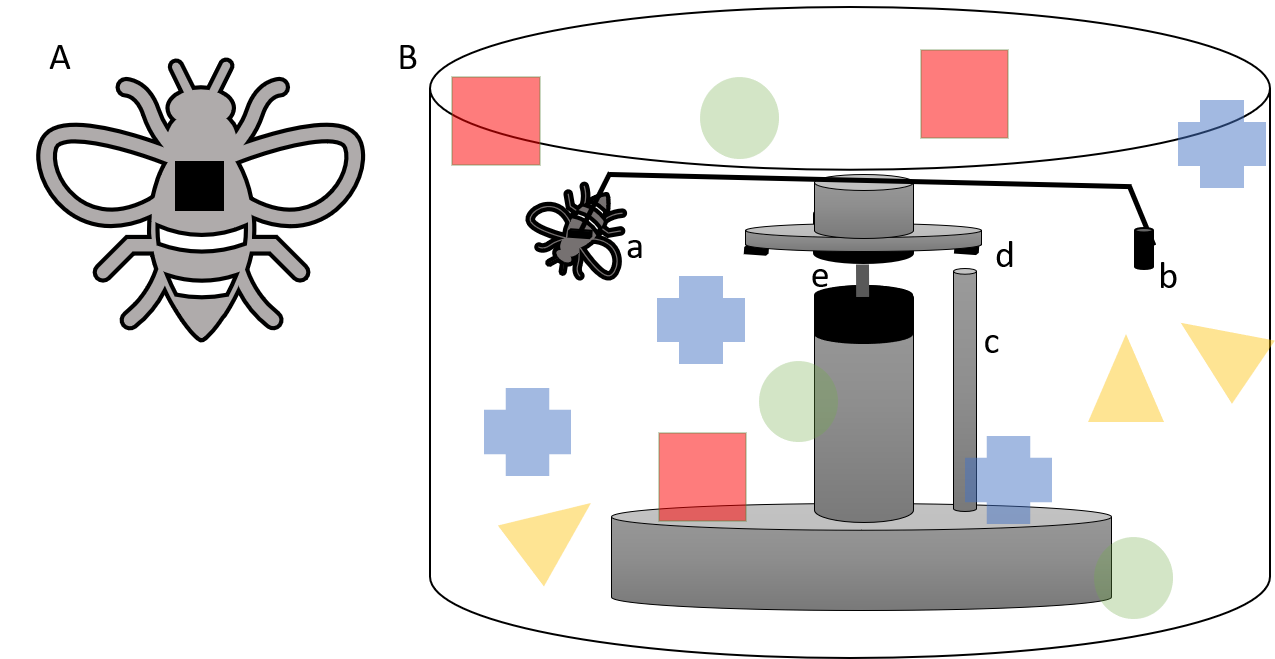


Supplementary Figure 3: A) Flying insect with a magnet tag on the thorax. B) Setup of the flight mill with a cylinder with visual cues and as wind protection (a = tethered insect, b = counterweight, c = Hall effect sensor, d = magnets on disc, e = magnetic rings).

# Supplementary Tables

## Supplementary Table 1

Overview of tagged and recaptured individuals and its condition.

| Number of individuals | total | poly | mono | *C. cyanus* | *P. tanacetifolia* | *S. arvensis* |
| --- | --- | --- | --- | --- | --- | --- |
| tagged | 413 | 141 | 272 | 91 | 93 | 88 |
| recaptured | 238 | 76 | 162 | 64 | 53 | 45 |
| without technical problems | 229 | 73 | 156 | 61 | 51 | 44 |
| initiated flight | 223 | 71 | 152 | 57 | 51 | 44 |
| tag position: ideal | 200 | 67 | 133 | 55 | 41 | 37 |
| tag position: unideal | 23 | 4 | 19 | 2 | 10 | 7 |
| tag position: unacceptable | 0 | 0 | 0 | 0 | 0 | 0 |
| with health problems | 17 | 9 | 8 | 4 | 3 | 1 |

Supplementary Table 2:

Data collection protocol: data that should be collected before tethering the insect and during the flight mill experiment.

| 1. **Initial data collected before tethering** | | |
| --- | --- | --- |
| 1 | ID information | Recording the colony or origin and sex |
| 2. | *Health status*: check for parasite infestation and damaged wings | Damaged or infested insects will be excluded from the experiment |
| 3. | *Tag* *position* on the thorax: ideal, unideal (not ideal in the centre of the thorax), unacceptable (very laterally glued to the thorax) (similar to the approach of Kenna et al. (2019) | The tag position data can be used to exclude unideal and/or unacceptable tagged individuals from experiment or for the analysis to test for the effect of different tag positions on flight performance |
| 4. | *Temperature* of the laboratory: the room temperature of the laboratory should be relatively constant during the experiment (around 25° C) (Kenna et al., 2021) | The temperature data can be used to test for the effect of temperature on flight performance |
| 5. | *Weight* of the individual and selection of counterweight | Each individual should be transferred into a small beaker that was closed with cotton wool. The beaker should be weighed with and without the insect with a fine scale to ascertain the weight of each individual. A counterweight can be selected on the basis of the determined weight and will be magnetically attached on the other end of the flight mill arm. The counterweight can be made out of modelling clay. For bumblebees we identified four suitable categories for counterweight in a preliminary study (200 mg, 250 mg, 300 mg and 350 mg).  The weight data can be used in the analysis to test for a relationship between body mass and flight performance |
| 6. | *Feeding* the individual with 50% sucrose solution | Cotton wool used for closing the beaker should be soaked with 50 % sucrose solution and offered to the insect as food. |
| 1. **Data collected after tethering** | | |
| 7 | *Number of tries to initiate flight* (we used a maximum of three: legs of the insect were touched with the landing platform and released) | Experiment was stopped if individual did not initiate flight after in our case three times helping with the landing platform |
| 8 | *Number of stops during flight* (we used a maximum of five times). When the insect stopped flying a landing platform should be offered for resting for 30 seconds and then removed again to stimulate flight | Experiment was stopped after certain number of flight stops or after certain time period. Number of stops per time unit can also be used as a measure of flight performance |
| 9 | *Body size* | After conducting the flight mill experiment, bee individuals can be again sedated and euthanized to enable body size measurements under the microscope e. g. inter-tegular span or wing size to test for covariation between body size and flight performance |
| 1. **Data collected with flight mill** | | |
| 10 | *Number of circuits/experiment*  *Linear velocity* (meters/second)  *Flight duration* | Maximum, mean and median of linear flight velocity during the experiment used as a measure of flight performance  Total flown distance during the experiment used as a measure of flight performance  Total flown time during the experiment used as a measure of flight performance |
| 1. **Data filtering** | |  |
| 11 | Deleting first flown circuit after a flight stop | Reason: the velocity is often extreme high in the first circuit comparted to the following circuits due to stimulatory stress |
| 12 | Deleting last three circuits before a flight stop | Reason: stopping the wing movements does not lead to an abrupt stop of the flight mill, but to a gradual slowing down |

Supplementary Table 3:

Quality and quantity of nectar and pollen of the investigated plant species (reviewed in Roulston et al. 2000 and Filipiak et al. 2022)

| Plant species | Total nectar/ flower [µg] | Total pollen/ flower [µg] | Protein content in pollen [%] |
| --- | --- | --- | --- |
| *Phacelia* *tanacetifolia* | 1040-9133 | 652 | 58.9 (genus *Phacelia*) |
| *Centaurea* *cyanus* | 420-720 | 350 | 26.2 |
| *Sinapis* *arvensis* | 516 – 664 |  | 33.8 |

Supplementary Table 4:

Overview of the timing of the tagging of the bumblebees and the measurements on the flight mill. Each tagging process 20 individuals were tagged in each mesocosm. For each flight mill experiment six tagged individuals were used.

| Diet | Mesocosm | Colony in mesocosm | First tagging proscess | Flight mill experiment | Flight mill experiment | Flight mill experiment | First tagging proscess | Flight mill experiment | Flight mill experiment | Flight mill experiment |
| --- | --- | --- | --- | --- | --- | --- | --- | --- | --- | --- |
| Mono Sa | D | 22.06.21 | 25.06.21 | 03.07.21 | 04.07.21 | 06.07.21 | 12.07.21 | 15.07.21 | 16.07.21 | 17.07.21 |
| Mono Sa | J | 22.06.21 | 29.06.21 | 03.07.21 | 04.07.21 | 05.07.21 | 07.07.21 | 15.07.21 | 16.07.21 | 17.07.21 |
| Mono Pt | E | 29.06.21 | 02.07.21 | 08.07.21 | 10.07.21 | 12.07.21 | 19.07.21 | 22.07.21 | 23.07.21 | 24.07.21 |
| Mono Pt | F | 29.06.21 | 06.07.21 | 09.07.21 | 11.07.21 | 12.07.21 | 14.07.21 | 22.07.21 | 23.07.21 | 24.07.21 |
| Mono Cc | B | 06.07.21 | 09.07.21 | 16.07.21 | 18.07.21 | 19.07.21 | 25.07.21 | 28.07.21 | 29.07.21 | 29.07.21 |
| Mono Cc | H | 06.07.21 | 13.07.21 | 17.07.21 | 18.07.21 | 19.07.21 | 20.07.21 | 28.07.21 | 29.07.21 | 30.07.21 |
| Poly | A | 06.07.21 | 13.07.21 | 18.07.21 | 20.07.21 | 21.07.21 | 25.07.21 | 30.07.21 | 02.08.21 | 03.08.21 |
| Poly | G | 06.07.21 | 14.07.21 | 18.07.21 | 20.07.21 | 21.07.21 | 22.07.21 | 30.07.21 | 02.08.21 | 03.08.21 |
| Poly | L | 06.07.21 | 11.07.21 | 18.07.21 | 20.07.21 | 21.07.21 | 23.07.21 | 30.07.21 | 02.08.21 | 03.08.21 |

Video 1: Laboratory set-up and carrying out measurements on the flight mill

Video 2: Anaesthetisation and bee tagging
